# Effect of glyceryl trinitrate and clevidipine administration on CT angiogram findings in dogs undergoing prostatic artery embolization for prostatic carcinoma

**DOI:** 10.1371/journal.pone.0316080

**Published:** 2024-12-27

**Authors:** Maureen A. Griffin, William T. N. Culp, Amandeep S. Chohan, Eric G. Johnson, Michelle A. Giuffrida, Carrie A. Palm, Robert B. Rebhun, Michael S. Kent

**Affiliations:** 1 Department of Surgical and Radiological Sciences, From the University of California-Davis, School of Veterinary Medicine, Davis, Davis, California, United States of America; 2 Department of Medicine and Epidemiology, University of California-Davis, School of Veterinary Medicine, Davis, Davis, California, United States of America; Private Urologist, HONG KONG

## Abstract

**Objectives:**

The primary aim of this study was to evaluate the effects of vasodilator administration on CT angiography (CTA) prostatic artery diameter and peak opacification in dogs with prostatic carcinoma prior to prostatic artery embolization (PAE).

**Materials and methods:**

A prospective clinical trial was performed. Ten dogs with naturally occurring prostatic carcinoma and no evidence of cardiovascular disease were enrolled. Each dog underwent multiphase CTA of the prostate before and after IV vasodilator (glyceryl trinitrate [GTN] or clevidipine butyrate [clevidipine]) administration, and cardiovascular parameters were monitored. PAE was performed the following day. Prostatic arterial anatomy was characterized by CTA. Prostatic artery lumen diameter and peak opacification were measured on pre- and post-vasodilator CTA by a blinded radiologist. The percent change of these measurements was calculated and assessed for significance.

**Results:**

Glyceryl trinitrate and clevidipine were administered in 5 dogs each with subsequent blood pressure reduction documented in all dogs. No significant difference was detected in prostatic artery diameter or peak opacification between pre- vs. post-vasodilator CTA. Good agreement in prostatic arterial branch number, origin, and course was documented between pre- and post-vasodilator CTA images.

**Clinical significance:**

Study findings do not support the routine use of vasodilator administration during pre-PAE CTA in dogs, though larger sample sizes and protocol alterations may be needed to detect a clinically relevant utility.

## Introduction

Prostatic artery embolization (PAE) has been well described for treatment of benign prostatic hyperplasia (BPH) in people [1,2]. This technique was initially demonstrated to be feasible, effective, and safe in both healthy research dogs and dogs with hormonally-induced BPH [3,4]. Recently, this technique has been adapted for treatment of dogs with spontaneously occurring prostatic carcinoma [5]. In a cohort of 20 dogs, clinical signs, characterized by tenesmus, stranguria, and lethargy, were significantly less common 30 days post-treatment compared to pre-treatment, and all dogs had a reduction in prostatic volume following PAE with a median of 39.4% volume decrease based on CT measurements [5]. In addition, the procedure was safe with no dogs experiencing major complications or postembolization syndrome [5]. Based on this information, PAE appears to be well tolerated and effective as a local treatment modality for prostatic carcinoma in dogs, though long-term outcome data is needed.

Although PAE is a promising minimally invasive local treatment for dogs with prostatic carcinoma, several challenges of this treatment modality are important to consider to optimize outcomes. For instance, the prostatic arterial supply is typically comprised of very small vessels (often 1–2 mm diameter in people and potentially smaller in dogs) with highly variable and complex anatomy, including the potential for vascular anastomoses with adjacent organs [6,7]. It is imperative to overcome these challenges and appropriately identify and select the prostatic arterial supply during PAE to both provide effective treatment and prevent non-target embolization. In humans undergoing PAE, CT angiography (CTA) has been described as an important tool that reliably predicts the prostatic arterial anatomy and facilitates PAE-planning [8]. Based on the established utility in humans, CTA is routinely performed prior to PAE in dogs with prostatic carcinoma.

Recently, additional techniques have been studied in people in an effort to enhance identification of the prostatic arterial supply on pre-PAE CTA. One study demonstrated that administration of sublingual glyceryl trinitrate (GTN) immediately prior to CTA resulted in a significant increase in prostatic arterial diameter and peak opacification [9]. Though this technique has not yet been evaluated in dogs with prostatic carcinoma, the effects of GTN and other vasodilators, such as clevidipine butyrate (clevidipine), on the canine cardiovascular system have been described in both anesthetized and conscious dogs [10–15].

The primary aim of our study was to evaluate the effect of vasodilator administration on pre-PAE CTA prostatic arterial diameter and peak opacification in dogs with naturally occurring prostatic carcinoma. We hypothesized that vasodilator administration would result in increased prostatic arterial diameter and peak opacification on pre-PAE CTA. A secondary objective of our study was to compare prostatic arterial imaging characteristics, such as branch number, origin, and course, between pre- and post-vasodilator CTA for dogs undergoing PAE for prostatic carcinoma. We hypothesized that characteristics of the arterial supply would be similar for pre- and post-vasodilator imaging studies.

## Methods

A prospective clinical trial was performed at the University of California-Davis, Veterinary Medical Teaching Hospital. Inclusion criteria were as follows: client-owned dogs with naturally occurring prostatic carcinoma diagnosed via cytology, histology, or positive urine BRAF mutational testing (Antech Diagnostics, Fountain Valley, CA); no evidence of cardiovascular disease based on history, physical examination, and resting non-invasive blood pressure measurement (with systolic pressure < 90 mmHg or mean arterial pressure [MAP] < 60 mmHg resulting in exclusion); and owners that elected PAE and pre-procedure CTA. Written informed client consent was obtained for each patient prior to enrollment. The study protocol was approved by the hospital’s Clinical Trials Review Board. Before enrollment, each dog was required to have pre-anesthetic clinical laboratory testing consisting of complete blood count and biochemistry panel and abdominal ultrasound to ensure there was no evidence of ureteral obstruction. Urinalysis, urine culture, and thoracic radiographs were also offered as pre-procedural staging diagnostics in all cases. To reduce the risk of contrast-induced nephrotoxicity and to maintain appropriate hydration and perfusion peri-anesthesia following enrollment, all dogs were maintained on IV isotonic crystalloids, (mL/kg/hr calculated by 70 x (body weight [kg])^0.75^/24hr) beginning several hours up to 1 day prior to CTA and ending 1 day following PAE. The CTA study and PAE procedure were both performed under general anesthesia separated temporally by 1 day.

### Anesthesia, CTA, and PAE protocols

For the pre-PAE CTA study, each dog was premedicated with 0.05 mg/kg hydromorphone IM except for one dog that instead received 0.2 mg/kg butorphanol IM. An appropriately sized intravenous catheter was placed in the cephalic vein after the required level of sedation was achieved. After preoxygenation, anesthetic induction was achieved with 4–6 mg/kg IV propofol titrated to achieve endotracheal intubation. After placing an appropriately sized endotracheal tube, anesthesia was maintained with isoflurane delivered in 100% oxygen. The patient was connected to a multiparameter monitor (Datex-Ohmeda; GE Healthcare, Chicago, IL) that recorded electrocardiogram, direct arterial blood pressure, end-tidal carbon dioxide and inhalant concentrations, esophageal temperature, and pulse-oximeter readings. A balanced isotonic crystalloid, Lactated Ringer’s Solution (LRS), was administered IV at a rate of 5 ml/kg/hr throughout the procedure. An arterial catheter was placed in the dorsal pedal artery to allow for arterial blood gas sampling and direct arterial blood pressure monitoring throughout the anesthetic event. A second intravenous catheter was placed in the lateral saphenous vein to facilitate contrast administration. Cardiovascular and respiratory parameters (heart rate, blood pressure, respiratory rate, oxygen saturation) were recorded at a minimum of every 5 minutes while each dog was anesthetized. All CT angiograms were performed with 0.6 mm slice thickness by a 16-slice CT scanner (GE Lightspeed; GE Healthcare, Chicago, IL) with dogs positioned in dorsal recumbency. Breath holds were utilized during CT angiograms to minimize motion.

To begin the CT study, a timing cine scan (5mm slice thickness) using a tracer dose of IV iopamidol (0.625 ml/kg Isovue 370 IV; Bracco Diagnostics, Inc., Princeton, NJ) was performed immediately prior to the two contrast CT studies to determine accurate CT timing for peak arterial enhancement. Cine CT was performed at the level of the aorta approximately 0.5 cm proximal to the aortic bifurcation. Peak aortic blush timing based on the cine CT imaging was used as the start time for the CTA. The cine contrast dose was delivered at 4ml/sec for timing. A dual phase angiography scan was then performed through the chosen anatomy with the remaining dose at 4ml/sec with an upfront delay calculated from the cine scan.

The two CTA studies were performed from 0.5 cm proximal to the aortic bifurcation caudally to the tuber ischii (arterial phase), then immediately back cranially to 0.5 cm proximal to the aortic bifurcation (mixed arterial/venous phase). For each CT angiogram, 1.2 ml/kg Isovue 370 was administered IV using a pressure injector immediately prior to imaging. Immediately prior to administration of the vasodilator, heart rate and blood pressure were recorded, and an arterial blood gas sample was obtained. This blood pressure measurement was used as baseline, and subsequent administration of vasodilator was performed to achieve a target MAP approximately 15 mmHg below the baseline measurement. Glyceryl trinitrate was administered either as an IV bolus (20 mcg/kg) or CRI beginning at a rate of 1–2 mcg/kg/min that was titrated to achieve the targeted MAP. Clevidipine was administered only as a CRI beginning at a rate of 1–2 mcg/kg/min and titrated until the targeted MAP was achieved. Immediately following the targeted MAP, an additional 1.2 ml/kg IV bolus of Isovue 370 was administered using a pressure injector, and the post-vasodilator CTA was performed using the same timing protocol and landmarks as the pre-vasodilator CTA. The two CTA studies were separated by at least 15 minutes to allow for washout of contrast material. If the vasodilator was still being administered during the CT scan, it was discontinued following this CT scan. Each dog’s heart rate and blood pressure were monitored throughout the study, and an additional arterial blood gas was obtained within 3–5 minutes following the second CTA study and discontinuation of the vasodilator CRI. Upon administration or titration of vasodilators, if any dog experienced hypotension characterized by MAP below 60 mmHg, it was managed by inhalant anesthetic delivery reduction, fluid bolus administration, and/or use of a dopamine constant rate infusion. All animals were monitored following general anesthesia, with cardiovascular parameters recorded within 24 hours of anesthesia and vasodilator administration.

One day following CTA, PAE was performed on each dog under general anesthesia by a single interventional radiologist (WTNC) with fluoroscopic guidance (OEC 9900 Elite; GE Healthcare, Chicago, IL) as previously described, with the goal of achieving vascular stasis to the prostatic arterial supply bilaterally [5].

### CTA measurements

**Fig 1:**
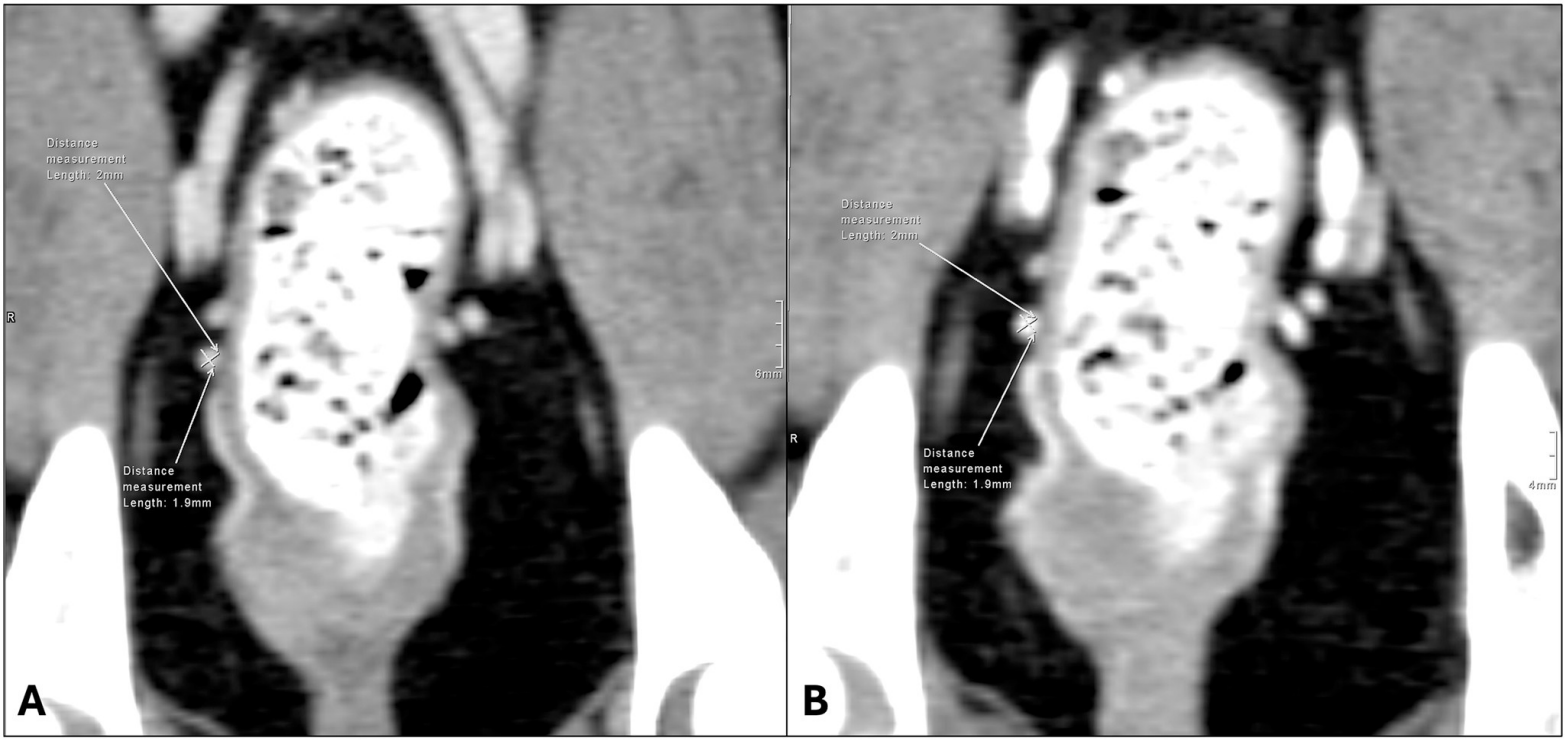
Measurements of prostate artery diameter pre-clevidipine (A) and post-clevidipine (B). The mean prostatic artery lumen diameter was taken from two orthogonal diameter measurements.

Following completion of the trial, all CTA images were analyzed independently by a board-certified radiologist (EGJ) who was blinded to patient details and whether the vasodilator had been administered. Initial image analysis was performed on a 3-megapixel diagnostic grade workstation, with images viewed via eFilm Workstation (Merge Healthcare, Chicago, IL). Measurements of prostatic arterial lumen diameter and peak opacification were performed using this dedicated workstation (Fig 1). Prostatic arterial lumen diameter was measured on the 0.6 mm axial images, at the point closest to the vessel origin that allowed clear measurement of vessel diameter (i.e., where the vessel was not abutting other vessels or high attenuation structures and was not running obliquely). If needed, images were reconfigured into 0.6 mm slices and a multiplanar reformatting was used to obtain accurate measurements. In all cases, the prostatic arterial lumen diameter measurement was performed within 1 cm of the origin to be deemed representative, since very little variation in vessel diameter occurs within the first centimeter.(9) The mean lumen diameter was taken from two orthogonal diameter measurements using the Profile function in the TeraRecon software (TeraRecon, Inc., Durham, NC). This function allowed for creation of an attenuation profile through a drawn line and was used to define points where attenuation reached three standard deviations below the mean. These were taken as the lumen wall to obtain a reproducible diameter measurement even in very small vessels. Peak lumen opacification values using Hounsfield units (HU) were also obtained from the same two vessel profiles, and the mean of the two measurements was recorded. Additional data that were recorded for each CTA study included number of prostatic arterial branches (external to the prostate), origin of prostatic arterial supply, and course of prostatic arterial supply. All data was recorded for both left- and right-sided prostatic arterial supply.

### Statistical analysis

A power analysis was performed. A sample size of 10 dogs was required to detect a 30% change in prostatic arterial diameter between measurements as significantly different from no change with 90% power (β = .10), assuming a standard deviation of 25% and α = .05. Descriptive statistics were calculated for patient and procedural variables. Percent change in diameter (mm) and peak contrast enhancement (HU) for each dog’s left and right prostatic artery branches measured at the level of their immediate bifurcation from the main prostatic artery before and after vasodilator administration was calculated according to the formula (PA_post_—PA_pre_)/PA_pre_× 100% and the population means were reported with surrounding 95% confidence intervals. Paired t tests were used to compare pre- and post-vasodilation arterial diameter, peak opacification, heart rate, MAP, and arterial blood gas variables. Unweighted kappa statistics were used to assess agreement between pre- and post-vasodilation arterial properties. P-value < 0.05 was considered statistically significant.

## Results

Ten dogs were included in the study. The following dog breeds were represented: 6 mixed breed, 1 Australian shepherd, 1 Labrador retriever, 1 Australian cattle dog, and 1 kelpie. All dogs were castrated males. The median age was 11.1 years (range 7.6–12.5). The median body weight was 24.7 kg (range 15.4–27.6).

Two dogs received a GTN IV bolus, 3 dogs received a GTN IV CRI, and 5 dogs received a clevidipine IV CRI; dosing information is provided in Table 1. Arterial blood pressure, heart rate, and various arterial blood gas parameters were recorded prior to and 5-minutes post vasodilator administration (Table 2). No significant difference was noted between any of these variables except MAP (P = 0.015). Two dogs that received a GTN IV bolus experienced an acute drop in MAP of 20 and 42 mmHg within 4 minutes post-injection. In the 3 dogs administered a GTN IV CRI, the MAP dropped by a median of 19 mmHg (range 15–21). In the 5 dogs administered a clevidipine IV CRI, the MAP decreased by a median of 15 mmHg (range 8–22). None of the dogs that received clevidipine CRI experienced any hypotension (MAP < 60 mmHg). One dog receiving GTN bolus and two dogs receiving GTN CRI required fluid boluses and use of a dopamine CRI in addition to reducing inhalant anesthetic delivery to manage hypotension. All dogs had a MAP > 60 mmHg within 5 minutes of vasodilator bolus injection or CRI discontinuation except one dog that received a GTN bolus and had a MAP of 53 mmHg. All dogs recovered uneventfully from general anesthesia, and cardiovascular parameters 1-day post-CT were within normal limits for all dogs; the median MAP for 7 dogs in which a resting blood pressure was obtained was 116 mmHg (range 92–147), and the median resting heart rate for all dogs was 97 (range 72–132). No intra- or post-procedural complications occurred with PAE for any dog, and all dogs were discharged 1-day post-PAE. Compared to pre-CTA/PAE labwork, initial post-PAE creatinine values (obtained 1–28 days post-PAE) were static to decreased in 9/10 dogs and minimally increased (by 0.1 mg/dL) in 1/10 dog.

**Table 1:** Vasodilator and dose information, including initial dose and dose at time of arterial blood pressure (MAP) reduction, as well as time between pre- and post-vasodilator CT angiogram scans in 10 dogs.

Vasodilator and route of administration	Number of dogs	Initial dose	CRI rate (mcg/kg/min) at time of MAP reduction: median (range)	Time (min) between pre- and post-vasodilator CT angiograms: median (range)
GTN IV bolus	2	20 mcg/kg	N/A	6 (1–11)
GTN IV CRI	3	1–2 mcg/kg/min	3 (2–9)	16 (15–24)
Clevidipine IV CRI	5	1–2 mcg/kg/min	4 (1–19)	13 (11–63)

**Table 2:** Arterial blood pressure (MAP), heart rate, and various arterial blood gas parameters in 10 dogs undergoing CT angiogram for prostatic artery embolization planning before (Pre) and 5 minutes after (Post) clevidipine or glyceryl trinitrate administration.

Parameter [Median (range)]	MAP (mmHg)	Heart Rate (Beats/min)	PaO_2_ (mmHg)	PaCO_2_ (mmHg)	pH	HCO_3_^-^ (mEq/L)	Lactate (mEq/L)
Pre	81 (68–131)	101 (65–164)	483 (406–569)	52 (40–69)	7.3 (7.2–7.4)	22 (21–26)	0.6 (0.4–1.4)
Post	71 (53–121)	118 (67–181)	498 (394–531)	48 (40–62)	7.3 (7.2–7.4)	22 (20–26)	0.6 (0.3–1.6)

In 1 dog, the left prostatic artery was not identified on either pre- or post-vasodilation CTA but was identified on fluoroscopic angiography. For all other dogs, both left and right prostatic arteries were identified on CTA and fluoroscopic angiography.

The mean arterial diameter and peak opacification for left and right prostatic arteries on pre- and post-vasodilator CTA is documented in Table 3. The mean percent change between pre- and post-vasodilator CTA left prostatic arterial diameter was +8.8% (95% CI 1.0% to 16.5%, P = 0.052). The mean percent change between pre- and post-vasodilator CTA right prostatic arterial diameter was +10.4% (95% CI -0.5% to 21.2%, P = 0.055). The mean percent change between pre- and post-vasodilator CTA left prostatic arterial peak opacification was -3.3% (range -50.0% to +96.9%, IQR -37.3% to +25.4%, P = 0.90). The mean percent change between pre- and post-vasodilator CTA right prostatic arterial peak opacification was +17.7% (range -53.4% to +90.2%, IQR -6.4% to +42.1%, P = 0.34). Therefore, neither prostatic arterial diameter nor peak opacification differed significantly between pre- and post-vasodilator CTA images for either left- or right-sided prostatic arterial supply.

**Table 3:** Mean arterial diameter and peak opacification for left and right prostatic arteries on pre- and post-vasodilator CTA.

	Left prostatic artery: pre-vasodilator CTA	Left prostatic artery: post-vasodilator CTA	Right prostatic artery: pre-vasodilator CTA	Right prostatic artery: post-vasodilator CTA
Mean diameter: mm (SD)	1.59 (0.26)	1.73 (0.41)	1.64 (0.11)	1.81 (0.29)
Mean peak opacification: HU (SD)	248 (61)	244 (136)	237 (73)	271 (107)

For the left prostatic arterial supply, there was good agreement between pre- and post-vasodilator CTA prostatic arterial origin (kappa 1.00, P<0.001). The left prostatic artery originated from the internal pudendal artery in 8 dogs, the internal pudendal artery but with anomalous branching in 1 dog and was not visible in 1 dog. There was 100% absolute agreement between pre- and post-vasodilator CTA left prostatic arterial origin. For the right prostatic arterial supply, there was good agreement between pre- and post-vasodilator CTA prostatic arterial origin (kappa 1.00, P<0.001). The right prostatic artery originated from the internal pudendal artery in 9 dogs and from a unique branch off the trifurcation of the internal iliac artery in 1 dog. There was 100% absolute agreement between pre- and post-vasodilator CTA right prostatic arterial origin.

For the left prostatic arterial supply, there was good agreement between pre- and post-vasodilator CTA prostatic arterial course in all 9 dogs in which it could be assessed (kappa 1.00, P<0.001). The left prostatic artery appeared straight in 1 dog, mildly tortuous in 4 dogs, moderately tortuous in 3 dogs, and looping or markedly tortuous in 1 dog. There was 100% absolute agreement between pre- and post-vasodilator CTA left prostatic arterial course. For the right prostatic arterial supply, there was good agreement between pre- and post-vasodilator CTA prostatic arterial course in all dogs (kappa 0.82, P<0.001). There was 90% absolute agreement in right prostatic arterial course between pre- and post-vasodilator CTA. For the 9 dogs with agreement, the right prostatic artery was mildly tortuous in 6 dogs, moderately tortuous in 2 dogs, and looping or markedly tortuous in 1 dog. For 1 dog, the right prostatic artery was looping/markedly tortuous on pre-vasodilator CTA and moderately tortuous on post-vasodilator CTA.

For the left prostatic arterial supply, there was good agreement between pre- and post-vasodilator CTA prostatic arterial branch number identified (kappa 0.71, P<0.001; absolute agreement 8/10, 80.0%). The left-sided branch number agreed for pre- and post-vasodilator CTA in 8 dogs: 1 dog had 0 branches, 2 dogs had 1 branch, 3 dogs had 2 branches, and 2 dogs had 3 branches. In the remaining 2 dogs, one had 2 left prostatic arterial branches on pre-vasodilator CTA and 3 branches on post-vasodilator CTA, and one had 3 left prostatic arterial branches on pre-vasodilator CTA and 2 branches on post-vasodilator CTA. For the right prostatic arterial supply, there was good agreement between pre- and post-vasodilator CTA prostatic arterial branch number identified (kappa 0.71, P<0.001; absolute agreement 9/10, 90.0%). The right-sided branch number agreed for pre- and post-vasodilator CTA in 9 dogs: 1 dog had 1 branch, 5 dogs had 2 branches, 2 dogs had 3 branches, and 1 dog had 4 branches. The remaining dog had 3 right prostatic arterial branches on pre-vasodilator CTA and 2 branches on post-vasodilator CTA.

## Discussion

In this clinical trial, we found no significant difference in prostatic arterial diameter, peak opacification, branch number, site of origin, or vascular course between pre- and post-vasodilator CTA images, despite achieving a targeted MAP reduction of ~15 mmHg in all dogs for the post-vasodilator CTA study. This finding calls into question the utility of vasodilator administration during pre-PAE CTA in dogs, as no significant effect was found. However, it is important to note that the sample size used in this study was based on a power calculation to detect a 30% difference in arterial diameter between groups. Therefore, this sample size is not adequate to find smaller percentage differences of significance. It is possible that a significant difference of < 30% change between groups may exist, and a greater sample size (based on a different power analysis) would be needed to identify this. However, the proportion of percent change or specific measurements for arterial diameter or peak opacification that are clinically relevant is not yet known. Moreover, a p-value of 0.052 and 0.055 was obtained for the percent change between pre-and post-vasodilator CTA images in the left and right prostatic artery diameters, respectively. It is possible that if a larger drop in MAP was targeted, a higher dose of vasodilators may have provided a larger degree of vasodilation resulting in a statistically significant (p < 0.05) change in the prostatic arterial diameter of the study dogs. Since these were client-owned dogs, a target drop of MAP by only 15 mmHg was adopted to minimize the occurrence of any clinically relevant hypotension. Also of note, although the mean arterial diameter was relatively increased post-vasodilator for both the left and right arterial supply, the mean arterial opacification was widely variable between pre- and post-vasodilator administration with a mean HU decrease for the left and mean HU increase for the right arterial supply post-vasodilator administration. In a human study, a significantly greater peak prostatic arterial opacification was identified in the group administered GTN compared to the group not administered GTN, but there was substantial overlap in these values including the 95% confidence intervals reported [9]. Thus, peak arterial opacification may be a less reliable parameter for assessment following vasodilator administration as compared to arterial diameter, though further studies are warranted.

Additional important findings of our study relate to vasodilator drugs and routes of administration in anesthetized dogs. In the absence of any hypertension, use of vasodilators in dogs under general anesthesia should be carefully monitored for any inappropriate drop in MAP. Hence, arterial catheters were placed in all the dogs to evaluate hemodynamic manipulation carefully, and CRIs of both GTN and clevidipine were initiated at low dose rates and titrated as required to achieve the targeted drop in MAP. Glyceryl trinitrate results in systemic arterial and venous vasodilation via rapid intracellular metabolism to its active metabolite nitric oxide, which subsequently promotes the production of intracellular cyclic guanosyl monophosphate resulting in smooth muscle relaxation [9,11]. Administration of GTN as an IV bolus resulted in an inconsistent drop in MAP with a variable timing to achieve desirable effect. In addition, the rate of return to normal blood pressure was also variable. Even when GTN was administered as a CRI in an effort to better titrate its administration to desired effect, an inconsistent CRI rate was required to achieve targeted MAP reduction. Three of five dogs (60%) that received GTN experienced hypotension and required fluid bolus and dopamine administration for management of hypotension. A potential adverse effect of GTN administration is methemoglobinemia [16,17]. Although direct measurement of methemoglobin was not performed in the present study, it is unlikely that the dogs experienced any clinically relevant methemoglobinemia since the total dose of GTN received by each individual dog was relatively low. In previous studies, dose administrations of GTN at which methemoglobinemia occurred include: IV CRI mean infusion rate 351 +/- 17 mcg/min for a total cumulative dose 3,398 +/- 308 mg in humans; PO 25 mg/kg/d for 5 days or 1 mg/kg/d for 12 months in dogs [16,17]. Clevidipine is a vascular selective L-type calcium channel antagonist of the dihydropyridine type [14]. The drug molecule has an ester linkage that undergoes rapid hydrolysis by plasma esterases resulting in ultra-short acting effect. Use of clevidipine in all five dogs resulted in a more controlled and consistent drop in the MAP as the dose was increased. Gross movement due to decreased anesthetic depth occurred in one of the dogs resulting in significant increase in heart rate and MAP while receiving clevidipine CRI, and this dog required a relatively larger dose (19 mcg/kg/min) to achieve target MAP. Based on our observations from the present study, it seems that clevidipine IV CRI is preferrable over GTN (IV bolus or CRI) for vasodilation to achieve controlled relative hypotension in dogs under general anesthesia.

Moreover, vasodilators can abolish hypoxic pulmonary vasoconstriction (HPV) which can increase ventilation-perfusion (V/Q) mismatching resulting in hypoxemia [18]. No significant change in the partial pressure of oxygen in the arterial blood (PaO_2_) was noticed in the present study. It is possible that breath holds employed during the imaging may have recruited some of the atelectatic lung regions, thereby offsetting any impact of increase in V/Q mismatching from the vasodilators. Cardiovascular depressant effects of various anesthetic drugs could result in decreased cardiac output (CO) and reduced oxygen delivery (DO_2_) to the tissues. Reduction in arterial blood pressure could further compromise blood flow through tissue beds. The arterial blood pressure post-vasodilator administration was significantly lower than pre-vasodilator measurement, suggesting that the impact of vasodilators was still present at 5-minutes post discontinuation. Although we did not measure CO and DO_2_ directly in these dogs, lack of any significant change in the levels of lactate suggests that tissues did not experience reduced oxygen delivery that may have resulted in type A hyperlactatemia in the study dogs.

Another finding of importance from this study is the highly variable nature of the prostatic arterial supply in dogs with prostatic carcinoma. In dogs of this cohort, the prostatic arterial size, peak opacification, and branch number varied substantially both between dogs as well as between left and right arterial supplies for each dog. This emphasizes the importance of thorough evaluation of angiographic studies in dogs with prostatic carcinoma, as individual variation is high, and these differences are important to recognize during PAE to ensure appropriate and effective embolization. Although this study identified good agreement between pre- and post-vasodilator CTA images for the origin, course, and number of branches of the prostatic arterial supply, the majority of dogs had discernable prostatic arterial anatomy on pre-vasodilator CTA. For more challenging cases in which the prostatic arterial anatomy is not clearly visualized on routine CTA, the utility of vasodilators to improve characterization of the prostatic arterial anatomy may be warranted and requires further evaluation. Additional information is needed in dogs with prostatic carcinoma to further assess the agreement between pre-procedural CTA and intra-procedural fluoroscopic angiography to determine the utility of CTA for PAE procedural planning.

There were several limitations of this study. First, there was a relatively small sample size, with even smaller subgroups for each vasodilator drug and route of administration. Because of the small sample size, comparisons between vasodilator drugs and routes of administration were not possible owing to the potential for error. In addition, this study was exploratory in nature and has provided important pilot data, but as such, it is possible that protocols other than those used in our methods could yield different results. Another limitation involves the inability to monitor methemoglobinemia in these dogs. Due to our inability to measure methemoglobin levels and inconsistent control of arterial blood pressure with GTN, we transitioned from use of GTN to clevidipine in this trial. Although an effort was made to standardize anesthetic drugs and perianesthetic management protocol, it is difficult to control all patient variables tightly in a clinical trial, and this may influence the outcomes. Another limitation to the study was using a 15-minute washout timeframe between dogs. This timeframe was chosen to minimize anesthetic time in client owned dogs. However, there likely was a small volume of residual contrast material in the vascular space of the dogs between studies. Despite this small residual intravascular contrast material, there was no significant difference in prostatic artery diameter or peak opacification between pre- vs. post-vasodilator CTA. Finally, an inherent limitation involves the image resolution with 0.6 mm slice thickness for the CTA studies relative to the small vessels of interest. Slight prostatic arterial variation within the 0.6 mm between slices may have occurred, with the potential to alter results of this study if these findings were to be captured with thinner CT slices.

In addition, although this data is useful in characterizing findings between pre-procedural CTA with and without vasodilator administration in dogs with prostate tumors undergoing PAE, the effect of vasodilator administration on procedural and clinical outcomes in dogs is not yet known. In humans and, anecdotally, in canine patients undergoing transarterial embolization of tumors, lidocaine can be administered intraarterially to both prevent/treat vasospasm of small vessels for subsequent embolization and also provide analgesia [19]. In rat models, vasodilators administered at the time of interventional procedures such as thermal ablation have been demonstrated to enhance treatment response [20]. Although the effects of IV vasodilator administration at the time of pre-procedural CTA are likely to be transient, additional study on any effect of vasodilator administration peri-procedurally relative to tumor response and clinical outcome is warranted in dogs with prostate tumors.

In conclusion, this prospective study is the first to evaluate vasodilator administration on CTA findings in dogs prior to embolization for naturally occurring tumors. The findings of this pilot study do not support the routine use of vasodilator administration for pre-procedural CTA in dogs undergoing PAE for prostatic carcinoma. However, a larger sample size with individual vasodilator drug and route of administration may be needed to detect significant differences. Based on the results of this study, if a vasodilator is used during CTA for anesthetized dogs, clevidipine, as an IV CRI titrated to effect, is recommended over GTN as an IV bolus or CRI. Future studies that characterize findings between CTA and PAE intraprocedural fluoroscopic angiograms for a large number of dogs are needed to determine the utility and sensitivity/specificity of pre-procedural CTA in dogs undergoing PAE for prostatic neoplasia.
